# Does patient behaviour drive physicians to practice defensive medicine? Evidence from a video experiment

**DOI:** 10.1186/s13561-023-00458-3

**Published:** 2023-09-22

**Authors:** Lotte Daniels, Wim Marneffe

**Affiliations:** Faculty of Business Economics, Hasselt University, Martelarenlaan 42, 3500 Hasselt, Belgium

**Keywords:** Defensive medicine, Medical liability, Video experiment, Patient behaviour, I11, I14, I18, K32

## Abstract

**Objective:**

By manipulating patients’ critical attitude in a video experiment, we examined whether physicians are more intended to perform defensive acts because of a higher perceived liability risk in Belgium.

**Methods:**

We assigned 85 practicing gynaecologists/obstetricians and orthopaedists randomly to four hypothetical video consultations, in which the patients show either a critical attitude (i.e., getting ahead of the facts, showing distrust) or a non-critical attitude (i.e., displaying more neutral questions and expressions). We asked the physicians about the care they would administer in the presented cases and the expected likelihood that the patient would sue the physician in case of a medical incident.

**Results:**

By manipulating patients’ verbal critical attitude (which indicates patients’ intention to take further steps), while keeping constant physician’s communication, patients’ clinical situation, preferences, and non-verbal behaviour in the videos, we were able to discover differential treatment styles driven by physicians’ perceived liability risk among patients with a different critical attitude. We found that physicians perform 17 percentage points more defensive acts (e.g., surgeries and diagnostic tests that are not medically necessary) when experiencing a high liability risk.

**Conclusions:**

Our results show that patients’ critical attitude drives physicians’ perceived liability risk and consequent defensive behaviour among obstetricians/gynaecologists and orthopaedists.

**Supplementary Information:**

The online version contains supplementary material available at 10.1186/s13561-023-00458-3.

## Introduction

By investigating and handling negligent caregiving, the medical liability system attempts to provide incentives to physicians to administer sufficient levels of care [1–3]. These incentives are predominantly driven not by indemnity payments but by the psychological, reputational and time costs associated with medical incidents and their unfolding, which are not covered by physicians’ malpractice insurances [4–7]. However, multiple empirical studies have indicated that the medical liability system may send confusing signals about the level of care physicians have to provide in order to escape liability, which may lead physicians to overestimate their medical liability risk and drive them to defensive medicine. Defensive medicine is defined as “the ordering of more tests, procedures and visits (assurance behaviour) or the avoidance of high-risk patients or procedures (avoidance behaviour), primarily (but not necessarily solely) to reduce the exposure to malpractice liability” [8, 9].

Although existing studies in this domain mainly focused on the impact of physicians’ legal working environment – for example, liability laws [3, 10–13], malpractice insurance premiums [14–16] – and physicians’ claim experience [17–19], physicians’ perceived liability risk is also expected to vary from patient to patient, which impacts the possibility of performing defensive acts on that particular patient.

To shed light on this issue, we conduct a video physician-experiment with patients’ critical attitude as a determinant of perceived liability risk. More specifically, we randomly assign practicing gynaecologists and orthopaedists to a sequence of four hypothetical video-taped consultations where the patient poses either neutral or critical questions, indicating patients’ intention to take further steps in case of malpractice. It is important to note that everything else in the videos is kept constant, including patients’ clinical situation, patients’ actions and physician communication. Furthermore, the patients’ critical attitude does not reveal additional information about the health status or preferences of the patient, so any difference in the outcomes is driven solely by a different level of criticism of the patient (that is, the way the patient verbally expresses her concerns). After watching each video, the physicians are asked to indicate which treatment(s) from a predetermined list of treatments they would prescribe (measure of treatment behaviour) and to demonstrate the extent to which they expect that each of the patients would sue the physician in case of an adverse event (measure of physicians’ perceived liability risk). We find that patients’ critical attitude drives physicians to more defensive treatment because of a higher perceived liability risk. More specifically, physicians are 17 percentage points more likely to perform defensive treatment when experiencing a high liability risk than when experiencing a rather low liability risk.

Our contribution to existing literature is twofold. First, we go beyond the state-of-the art of defensive medicine literature by using an experimental design to assess whether the fear of medical malpractice claims affects physicians’ level of care, keeping constant all other factors that may impact the level of care, such as severity of the condition or patients’ appearance. Many results in existing studies suffer from confounding because of insufficiently fine-grained observational data; this is not a problem in our experimental design because of two-stage randomisation and controlled manipulation.

Second, we advance health care discrimination literature by investigating physicians’ perceived liability risk as an explanation for discriminating physicians’ behaviour. We are able to measure agency discrimination by varying only the manner of questioning of the patient (either critical or neutral) indicating physicians liability risk, keeping constant patients’ explanation of symptoms, clinical indications, what the physician says, nonverbal behaviour, etc. This research differs from other studies in the field that mainly focus on discrimination as such (and are therefore unable to assess the drivers of discrimination; e.g., Schulman et al. [20]) and those that solely address statistical discrimination (e.g., Balsa and Mcguire [21]; Grytten, Skau, and Sørensen [22]) or other drivers of agency discrimination, such as financial incentives (e.g., Johnson and Rehavi [23]; Milcent and Zbiri [24]).

Sect. " Related literature" discusses the related literature and Sect. " Design and methods" clarifies the experiment set-up. Our descriptive and empirical results are presented in respectively Sect. " Descriptive Statistics" and " Econometric Analysis and Results", and discussed in Sect. " Discussion and Limitations". Sect. " Conclusion" concludes.

## Related literature

To explore the prevalence of defensive medicine, it is necessary to analyse the impact of physicians’ perceived liability risk on the level of care. An initial approach is to directly ask physicians about their perceived liability risk and analyse how this affects their treatment behaviour. Although such surveys are common (e.g., Carrier, Reschovsky, Katz, and Mello [25]; Reschovsky and Saiontz-Martinez [26]), they use physician-level data and hence do not control for patient characteristics, nor can they take into account the fact that physicians’ perceived liability varies from case to case. The OTA study [9] addressed these concerns using hypothetical clinical scenarios (paper-based), but lacks a strong manipulation and external validity.

Second, scholars exploited changes in the determinants of perceived liability risk. Research in this field mainly focused on changes in the structural working environment of physicians – for example, liability laws [3, 10, 12, 13] and malpractice insurance premiums [14–16]. However, these changes are possibly endogenous because they could also be driven by suboptimal levels of care; for example, claim frequency and severity drive changes in insurance premiums and liability laws and also in the level of care. Finding the true causal effect using observational data requires sufficiently fine-grained data on confounders that affect the level of care and drive these changes at the same time.

Furthermore, physicians’ overall perceived liability risk may increase because of patients’ suing behaviour. For example, researchers used cross-sectional and panel data on physician level to assess the impact of their experience with malpractice claims on C-section rates [18, 27]. Other researchers have exploited the exact timing of procedures in an event study to analyse whether these events lead to a discontinuous increase in treatments and whether this is a temporary or long-lasting effect. In their event studies, Shurtz [19] and Dranove and Watanabe [17] found that a malpractice lawsuit impacts C-section rates, but only temporarily. Both studies also analysed whether physicians change their behaviour in the same manner when peers encounter a lawsuit.

Besides focusing on overall increased liability risk due to, for example, stricter liability laws or claim experience, one can look at the impact of particular patient characteristics that are associated with likelihood to sue. For example, various researchers have shown that elderly, poor, and uninsured patients are less likely to file malpractice claims (e.g., Burstin, Johnson, Lipsitz, and Brennan [28]). However, McClellan, Jimenez, and Fahmy [29] indicated that physicians incorrectly assume that socioeconomically disadvantaged patients tend to sue more frequently. Studies indicating that patients’ characteristics such as race, sex, insurance or expertise are associated with different treatment styles [20, 22, 23, 30] have raised the question of whether a difference in perceived liability risk is the underlying cause of differential treatment styles.

To date, the literature has described two potential explanations for discrimination in health care: (1) statistical discrimination (that is, the physician makes the best choice possible based on the information s/he has – for example, physicians may treat certain patients better because they are better at communicating symptoms with the physician [21, 22]), and (2) agency discrimination (that is, there is a conflict of interest between the physician and the patient and the physician may exploit the information advantage s/he has—for example, physicians may persuade patients with a low expert level to receive specific treatments because of financial incentives [23, 24, 31–34]. We consider defensive medicine as a form of agency discrimination, as physicians, in case of asymmetric information, may let their self-interest rule and practice defensively to reduce their personal liability risk, driven, for example, by patients’ critical attitude. However, papers about agency discrimination have focused mainly on physicians’ financial incentives, and not on defensive medicine. The only evidence in this field is the results of the survey by Komaromy, Lurie, and Bindman [35], which indicated that the perception of an increased risk of being sued is a decisive factor for physicians in their clinical decision-making for patients with a lower social status. However, physicians may be implicitly and unconsciously influenced, which is not uncovered by inquiring physicians directly. Uncertainty remains regarding the extent to which patient characteristics and behaviour drive physicians to practice defensive medicine.

## Design and methods

To investigate whether physicians prescribe more defensive treatment when experiencing high liability risk driven by patients’ critical attitude, we conducted a video-experiment in which actual gynaecologists and orthopaedists were asked to indicate which treatments they would prescribe in four hypothetical video consultations (recorded in an actual consultation room with actual physicians and patients). At the same time, we asked physicians to indicate their perceived liability risk with every patient. To create enough variety in physicians’ perceived liability risk, we exposed half of the physicians to a version of the videos with a critical patient, while the other half was confronted with exact the same videos, except that the patient shows a more neutral attitude. Keeping constant all other factors in the videos, such as physician communication and the patient’s health status, preferences and non-verbal behaviour, allows us to assess the causal impact of being a critical patient on physician clinical behaviour through a higher perceived liability risk.

### Scenarios

Each physician evaluated four videos, each regarding a hypothetical consultation of their own specialisation (for example, gynaecologists only saw the four gynaecologist cases). In particular, the videos concern a summary of symptoms by the patient and a question–answer interaction between the patient and the physician. We included cases pertaining to obstetrics/gynaecology and orthopaedics because of the relative high liability risk exposure in these specialties [36, 37]. The former is the most researched speciality when it comes to defensive medicine, but empirical research regarding other specialities is scarce.

The videos are about risk procedures. For routine consultations, the perceived liability risk would always be close to zero. In order to objectively measure physicians’ treatment behaviour in the presented cases, the physician–patient interaction in the videos stops after all relevant clinical indications are explained, and before the physician in the video expresses his treatment preferences. Also, we did not use names and demographics other than those demonstrating patients’ clinical situation. The duration of the videos (maximum 5 min), was considered realistic according to initial discussions with gynaecologists and orthopaedists.

In order to obtain realistic and valid scripts and manipulations, we conducted a thorough literature study and closely collaborated with 36 professionals^1^ who had expertise in patient safety, communication and medical liability in the Flemish health care context. Since we are interested in the extent to which patient behaviour drive physicians to practice defensive medicine, we manipulated the critical attitude of patients in the videos. Important to note is that we did not explicitly implement the case of being sued and the potential consequences in the videos. We trigger physicians’ perceived liability risk rather implicitly by varying patients’ critical attitude, as a result of which physicians might fear the potential financial, reputational and moral costs in case of a medical incident.

We have two versions of each case: one where the patient demonstrates a critical attitude (treatment case) and one where the patient shows a rather non-critical or neutral attitude (control case). Together with the expert panel, we defined differences between the two versions into the following patient behaviours: (1) making assumptions and get ahead of the facts, (2) asking for guarantees, (3) showing distrust, and (4) showing knowledgeability about the medical situation and possible consequences. After writing draft scenarios, several experts from our panel gave remarks and made adjustments until consensus was reached regarding the validity and realism of the scripts. Table 1 shows the manipulations for the case of a 38-week pregnant women whose firstborn was delivered by C-section. Full transcripts of the videos of this case can be found in the Appendix. Transcripts of the other videos can be requested from the author.

**Table 1:** Manipulations scenario previous c-section

Manipulated element	Non-critical patient	Critical patient
Questions posed	Neutral questions *Example: Eventually, suppose I’ll give birth by C-section, would that be an emergency procedure or how should I prepare for that? For example, in that case, would it be possible for my husband to attend the delivery?*	Making assumptions and get ahead of the facts *Example: If I understand correctly, I still have a 25 per cent chance that I’ll get an emergency C-section and that my husband cannot attend the delivery because I’ll get anaesthesia*
Reaction to answers of physician	Understanding *Example: Okay, I can imagine. And would that be dangerous for the baby or wouldn’t you expect any complications?*	Asking for guarantees *Example: But can you guarantee that there will be no oxygen deprivation for the baby and that he will not suffer? I hope you don't take any risk that could harm me or the baby*
Trust	Showing trust *Example: Okay, I understand. You probably have experience with this*	Showing distrust *Example: But do you know how to respond? Do you already have experience with such situations?*
Knowledgeability about medical situation and possible consequences	Nothing mentioned about level of information	Showing that the patient is informed *Example: My neighbour is also a gynaecologist and he told me that such a tear can bleed dramatically so that I could lose my uterus*

Everything besides the manipulations is held constant, including patients’ health status, patients’ actions, the consultation room, and physicians’ communication. For example, the physicians’ communication in the videos was recorded once (only audio) and edited in the two versions of patients’ communication, so the only difference in the two versions of a given scenario is in the patients’ communication. At the same time, we closely monitored patients’ non-verbal communication during recording. While the first participant watched a consultation in which the patient shows a greater verbal critical attitude, we asked another participant to watch the exact same consultation, with the same patient, the same actions, except that the patient did not have a verbal critical attitude. In that way, we could rule out non-verbal behaviour (such as facial expressions, tone of voice) to bias the effect of patients’ verbal critical attitude. Another argument to support the assumption that we have arguably perfect counterfactuals is that the patients’ critical questions do not reveal additional information about the health status or preferences of the patient. In that way, we ensure that any difference in the outcomes is driven by a different verbal attitude of the patient and not by, for instance, more severe clinical indications or other preferences.

### Production of the videos

We were assisted by a professional production firm to guarantee high-quality sound and vision in the videos. We used a static camera that was set at the physicians’ point-of-view so that participants only saw the patient in the consultation room when watching the videos (as it would be in a genuine consultation). To ensure realistic interactions, an actual male physician role-played the physician in the videos.^2^ We searched for people who matched the patients’ characteristics in the scripts to act as patients in the videos (for example, a 38-week pregnant woman for the former C-section case). We asked the physicians and the patients to practice their role a priori. After recording multiple tapes of each version, we asked multiple physicians to select the most realistic tapes for the final pilot-check. A real consultation room was used as a setting to produce the videos.

### Manipulation checks

In order to get realistic videos with strong manipulations, we asked our expert panel to give feedback on both the scripts and the videos. Van Vliet, Hillen, Van der Wall, Plum, and Bensing [39] stated that a double pilot-check is very important since videos may be perceived differently than scripts and may, therefore, ask for different adjustments. Moreover, a second pilot test can be used to test the effect of first adjustments. We asked another six physicians to validate the realism of the scenarios and the questions and scales that we used in the experiment. After minor adjustments in terminology, our videos and questionnaire were deemed valid and realistic for our research purposes.

To check whether our manipulations were perceived as intended by the final participants, we added some manipulation checks in our questionnaire. More specifically, participants were asked to rate, on seven-point Likert scales, the following patient characteristics and behaviours: (1) trusting physician, (2) having prior medical knowledge, (3) anticipating of facts, (4) suspiciousness, (5) critical attitude, (6) concern, (7) anxiety, (8) friendliness, (9) politeness, and (10) calmness. All of these characteristics were perceived significantly different between the two versions in a way we intended (significance level of 1 per cent), except for the level of prior medical knowledge (see Table 2). This means that participants perceived the critical patient as showing less trust in the physician, more anticipating of facts, being more suspicious, critical, concerned, anxious, less friendly, polite, and calm. Although the critical patient is perceived as more knowledgeable about medical conditions and potential consequences a priori, this difference is not significantly different from zero (*p*-value = 0.222).

**Table 2:** Manipulation checks

	Mean		
	Control group mean (1)	Treatment group mean (2)	OLS Difference (3)
*Patient Characteristics/Behaviours in Videos*			
Trusting Physician	5.58	3.54	-2.04*** (0.000)
Having Prior Medical Knowledge	3.79	4.11	0.33 (0.222)
Anticipating of Facts	3.08	4.64	1.56*** (0.000)
Suspiciousness	2.78	5.04	2.27*** (0.000)
Critical Attitude	4.57	5.32	0.75*** (0.000)
Concern	4.91	5.94	1.03*** (0.000)
Anxiety	3.21	4.99	1.77*** (0.000)
Friendliness	6.02	4.87	-1.16*** (0.000)
Politeness	6.18	5.28	-0.89*** (0.000)
Calmness	5.78	4.80	-0.98*** (0.000)
*Physician Characteristics/Behaviours in Videos*			
Experience	5.64	5.28	-0.37 (0.150)
Capability	5.45	5.33	-0.12 (0.608)
Taking Time	5.88	5.87	-0.00 (0.993)
Handling Properly	5.43	5.18	-0.24 (0.376)
Giving Enough Information	5.43	5.47	0.04 (0.876)
Giving Correct Information	5.43	5.38	-0.05 (0.854)

The table reports the results based on manipulation checks. All variables were measured on seven-point Likert scales. Columns (1) and (2) display means for the control and the treatment group, respectively. Column (3) reports the coefficients from an OLS regression with *Critical Patient* as the explanatory variable, with corresponding *p*-values shown in (parentheses). We clustered heteroskedasticity robust standard errors at the participant level

At the same time, the physicians in the videos were perceived as equally (1) experienced, (2) capable, (3) taking time for the patient, (4) handling properly, (5) giving enough information to the patient, and (6) giving correct information in the two versions. This supports the assumption that the non-critical patient videos are good counterfactuals for the ones demonstrating critical patients (only the manipulated elements are driving the treatment effects).

### Sample and procedures

The sample consisted of practicing specialists within obstetrics/gynaecology and orthopaedics (each physician saw four cases regarding their own specialty). We controlled for incident experience of the physicians but did not pose any exclusion criteria on that. Also, we carefully selected general cases so that no further specialisation (for instance, knee orthopaedist or oncologist-gynaecologist) was needed in order for the physicians to be able to evaluate the videos. Based on a power analysis with an anticipated effect size of 0.15, a desired statistical power level of 0.90 and a probability level of 0.05, the anticipated sample size was at least 150 observations. Given that we want to estimate treatment effects for obstetricians/gynaecologists and orthopaedists separately in interaction terms, and given that each physician sees four videos, we needed at least 30 of each. To find a sufficiently large sample, we contacted various professional physician associations and used mailing lists of all Flemish hospitals. Data were collected between February 2022 until May 2022.

Participants were randomly assigned to a sequence of four videos, each regarding a hypothetical medical consultation with either a critical or non-critical attitude of the patient.^3^ Participants saw four videos with the same manipulations in a between-design (for example, while the first physician saw four videos with a critical patient, another physician watched the same videos but with a non-critical patient). The videos were shown in random order to control for order effects.

After watching each video, the physician was presented with a predetermined list of treatments (drafted by the expert panel and checked with physicians) and asked to indicate which one(s) s/he would prescribe (measure of treatment behaviour).^4^ Crucially, we also asked about the perceived likelihood that each of the patients would sue the physician in case of an adverse event, indicating physicians’ perceived liability risk. At the end of the experiment, we asked the physicians to fill out a questionnaire to elicit information about, for example, their demographics, working environment and experience, experience with adverse events, and overall defensive nature.^5^

To minimise the time that physicians had to spend on the experiment, the experiment was conducted online. The average duration of the experiment was 25 min.

### Key variables

Table 3 shows the definitions of the key variables. Our main outcome is *Defensive Treatment*, which is a dummy equal to one if the physician prescribes defensive treatment for the case presented in the video. We made distinctions between defensive and non-defensive treatment through thorough discussions with our expert panel. Examples of defensive treatment in the pre-determined list are C-sections, labour inductions or extra ultrasounds in the gynaecological cases and unnecessary scans and surgeries in the orthopaedic cases. In the presented cases, experts considered these procedures not strictly medically necessary and even harmful in some instance (e.g., excessive radiation exposure, more invasiveness). Since they, however, provide the physicians more feelings of control to avoid medical incidents and/or evidence to cover themselves in case of a medical incident, they are considered defensive (e.g., a C-section is one of the most described defensive practices in literature, as the failure to perform such a practice in a timely C-sections manner is one of the most common accusations in malpractice claims against gynaecologists and obstetricians). Important to note is that in the Belgian fee-for-service payment structure [40], these defensive treatments also result in higher revenues for the physicians, which may further drive physicians to practice defensive medicine, besides lowering liability risk.^6^ Our variable of interest is physicians’ perceived liability risk, which is a dummy equal to one if the respondent gave a mean score higher than the scale centre of four on a seven-point Likert scale to the extent that (s)he expects the patient would take further steps in case of a medical incident. We also controlled for physicians’ demographics, working and legal background.

**Table 3:** Variable definitions

Variable Name	Description
*Outcome*	
Defensive Treatment	Dummy equal to 1 if physician chooses defensive treatment
*Variable of Interest*	
Liability Risk	Dummy equal to 1 if mean physician’s perceived medical liability risk is greater than or equal to 4 on a seven-point Likert scale.^a^
*Treatment Variable*	
Critical Patient	Dummy equal to 1 if physician saw videos with critical patient communication
*Physician Characteristics*	
Female	Dummy equal to 1 if physician is female
Gynaecology	Dummy equal to 1 if physician saw gynaecological cases and 0 when exposed to orthopaedic cases
Working Experience > 20y	Dummy equal to 1 if physician practices medicine for more than 20 years
Accountability	Dummy equal to 1 if physician is accountable to a superior/colleagues for the actions (s)he takes
Claim Experience	Dummy equal to 1 if physician has personal claim experience
Defensive Person	Dummy equal to 1 if the mean of 10 defensive acts (that is, lowering patient contacts, treating less high-risk patients, performing less high-risk treatments, working less hours, retiring early, changing to a lower risk specialism, referring patients to confirm diagnosis (second opinion), prescribing more medication, suggesting more diagnostic procedures to confirm diagnosis, suggesting more invasive tests to confirm diagnosis) is greater than or equal to 3

^a^How likely do you think it is that the patient would take further steps against the treating physician if problems or complications would arise as a result of medical treatment? (1 = very unlikely, 7 = very likely)

## Descriptive statistics

Eighty-five physicians (48 gynaecologists and 37 orthopaedists) evaluated all videos in the final experiment. After removing the cases for which participants did not specify other treatments than the ones in the list, we have a focal sample of 328 observations. The descriptive statistics of our sample are presented in Table 4. Most of the participants were male (65 per cent). Approximately half of the sample were gynaecologists (56 per cent), had working experience of more than 20 years (48 per cent) and reported previous claim experience (49 per cent). About two-fifths are accountable to a superior or peer (40 per cent) and are defensive in nature (40 per cent).

**Table 4:** Descriptive statistics

Variable Name	Obs	Mean	Std. Dev	Min	Max
*Outcome*					
Defensive Treatment	328	0.34	0.47	0	1
*Variable of Interest*					
Liability Risk	328	0.48	0.50	0	1
*Treatment Variable*					
Critical Patient	328	0.48	0.50	0	1
*Physician Characteristics*					
Female	85	0.35	0.48	0	1
Gynaecology	85	0.56	0.50	0	1
Working Experience > 20y	85	0.48	0.50	0	1
Accountability	85	0.40	0.49	0	1
Claim Experience	85	0.49	0.50	0	1
Defensive Person	85	0.40	0.49	0	1

Thirty-four per cent of the participants prescribed defensive treatment after the consultations, which is our primary outcome. Almost half of the sample (48 per cent) perceived a relatively high liability risk.

For a particular case, physicians were randomly assigned to either the critical or non-critical version; therefore, physician characteristics between treatment and control groups should be similar. To check this assumption of balance, we ran an OLS regression per physician characteristic with *Critical Patient* as explanatory variable. Table 5 shows the results of these OLS series. No significant differences were found in the participant characteristics between the treatment and control groups.

**Table 5:** Results on tests of covariate balance

	Mean		
	Control group mean (1)	Treatment group mean (2)	OLS Difference (3)
*Physician Characteristics*			
Female	0.34	0.37	0.02 (0.813)
Gynaecology	0.52	0.61	0.09 (0.424)
Working Experience > 20y	0.48	0.49	0.01 (0.924)
Accountability	0.41	0.39	-0.02 (0.861)
Claim Experience	0.48	0.51	0.03 (0.751)
Defensive Person	0.41	0.39	-0.02 (0.861)

*Notes: *The table reports the results based on tests of covariate balance. Columns (1) and (2) display means for the control and the treatment group, respectively. Column (3) reports the coefficients from an OLS regression with *Critical Patient* as the explanatory variable, with corresponding *p*-values shown in (parentheses)

Table 6 provides descriptive statistics of our outcome and variable of interest for our control and treatment group, respectively. We see that physicians practice more defensively and experience a higher liability risk with a more critical patient. These differences are significant at the 10 per cent and 1 per cent significance levels, respectively.

**Table 6:** Descriptive statistics defensive treatment and liability risk

	Non-Critical Patient (1)	Critical Patient (2)
(1) Defensive Treatment	0.30* (0.04)	0.39* (0.04)
(2) Liability Risk	0.27*** (0.03)	0.72*** (0.04)
N	169	159

Cells contain means and (standard errors). *** *p* < 0.01, ** *p* < 0.05, * *p* < 0.1

## Econometric analysis and results

### Empirical strategy

To investigate whether patients’ critical attitude drives physicians’ perceived liability risk and consequent defensive behaviour, we estimated Eq. (1).

1 $${Y}_{ic}={\alpha }+\beta {Critical Patient}_{ic}+\gamma {{Liability Risk}_{ic}+{X}_{i}\Theta +\varphi }_{c}+{\varepsilon }_{ic}$$

${Y}_{ic}$ is a dummy equal to one if physician *i* prescribes defensive treatment for consultation *c*. ${Critical Patient}_{ic}$ indicates whether physician *i* sees the critical patient version of case *c,* and ${Liability Risk}_{ic}$ the perceived liability risk.^7^$\gamma$ is the estimated effect of liability concerns on prescription behaviour, and $\beta$ the estimated effect of patients’ critical attitude. ${\varphi }_{c}$ are case fixed effects. Alternatively, we replaced case fixed effects with a dummy indicating whether the physician watches gynaecological or orthopaedic cases (*Gynaecology*). This allowed us to examine whether defensive medicine is more or less prevalent among gynaecologists in comparison to orthopaedists. We also controlled for some other physician characteristics (vector ${X}_{i}\Theta$). This would be important in the analysis of observational data since physicians may embody attributes that confound the results (for example, physicians with claim experience may administer more defensive acts). This should not be a concern in our research because, by design, patients’ critical attitude is orthogonal to case and physician characteristics. Nevertheless, we control for physicians’ gender, accountability, working and claim experience, and overall defensive nature.^8^ We cluster standard errors at the physician level and add order fixed effects to avoid bias from order effects.

### Main results

Table 7 shows the main results of Eq. (1). In Row (1), we only include our treatment variable (*Critical Patient*) and controls, and not physicians’ perceived liability risk. Comparing these coefficients with those of Row (2), in which we additionally control for perceived liability risk, allows us to uncover whether physicians perform more defensive treatment when confronted with a critical patient because of a higher perceived liability risk or because of other factors that may be related to a more critical patient (for example, a critical patient to be more persuasive). Particularly, if physicians’ perceived liability risk would drive defensive treatment (and not other factors related to a more critical patient), our variable *Liability Risk* would absorb the treatment effect so that being a critical patient would not have an additional significant impact (besides the impact of physicians’ perceived liability risk). Our results show this is indeed the case.

**Table 7:** Main results

	Dependent Variable: Defensive Treatment			
	(1)	(2)	(3)	(4)
Critical Patient	0.111**	0.036	0.056	0.057
	(0.052)	(0.057)	(0.055)	(0.055)
Liability Risk		0.171***	0.128**	0.127**
		(0.062)	(0.057)	(0.057)
Female	-0.122*	-0.134**	-0.133*	-0.136**
	(0.070)	(0.067)	(0.067)	(0.067)
Working Experience > 20y	0.107*	0.090	0.097	0.097
	(0.059)	(0.061)	(0.059)	(0.060)
Accountability	0.059	0.073	0.068	0.069
	(0.056)	(0.057)	(0.057)	(0.057)
Claim Experience	-0.023	-0.017	-0.026	-0.027
	(0.067)	(0.069)	(0.068)	(0.068)
Gynaecology		-0.053		
		(0.110)		
Defensive Person				0.017
				(0.057)
Observations	328	328	328	328
Case FE	Yes	No	Yes	Yes
Order FE	Yes	Yes	Yes	Yes

The table reports OLS results. Heteroskedasticity robust standard errors in (parentheses) are clustered at the participant level. *** *p* < 0.01, ** *p* < 0.05, * *p* < 0.1

For example, in Column (1), we see that physicians watching the critical patient videos act significantly more defensively than those encountering a non-critical patient. In particular, critical patients receive more than 11 percentage points more defensive treatments than their non-critical counterparts. This effect becomes insignificant when adding physicians’ perceived liability risk, which our results show has a highly significant impact on our outcome [Column (2)]. Therefore, the significant result of critical patient in Column (1) is absorbed by the significant impact of physicians’ perceived liability risk in Column (2). These findings provide evidence that the effect of being a critical patient is not driven by factors other than liability risk (otherwise, the coefficient of critical patient would remain significant in addition to the coefficient of liability risk). In sum, physicians who perceive a higher liability risk performing more than 17 percentage points more defensive acts. Our results are consistent when replacing the gynaecology dummy with case fixed effects in Column (3). Additionally, Column (4) shows that our results are not influenced by adding a variable indicating the overall defensive nature of physicians. Also, being defensive by nature is not associated with significantly more defensive acts. However, our results suggest that women practice significantly less defensive medicine and that physicians with more working experience are intended to perform more defensive acts.

### Interacting effects

We also examine possible interaction effects in Table 8. First, between physician characteristics and the impact of (1) patients’ critical attitude [Columns (1–3)] and (2) perceived liability risk on physicians’ behaviour [Columns (4–6)]. Second, between the impact of liability risk on physicians’ behaviour and patients' critical attitude [Column (7)]. After all, physicians with certain characteristics (for example, gynaecologists, women, and those who are accountable to peers or superiors) may be more or less impacted by patients’ critical attitude and consequent perceived liability risk. The results show that none of these interaction effects are significantly different from zero. In other words, the effect of consulting a critical patient or experiencing a high liability risk is similar among subgroups regarding to specialism, gender and accountability.

**Table 8:** Results with interaction terms

	Dependent Variable: Defensive Treatment						
	(1)	(2)	(3)	(4)	(5)	(6)	(7)
Critical Patient	0.001	0.060	0.059	0.039	0.058	0.057	0.127
	(0.096)	(0.076)	(0.074)	(0.060)	(0.055)	(0.055)	(0.079)
Liability Risk	0.176***	0.127**	0.127**	0.149	0.094	0.122	0.200**
	(0.065)	(0.057)	(0.057)	(0.102)	(0.074)	(0.079)	(0.077)
Female	-0.137**	-0.132	-0.136**	-0.137**	-0.189**	-0.135*	-0.132*
	(0.066)	(0.085)	(0.068)	(0.066)	(0.091)	(0.068)	(0.067)
Working Experience > 20y	0.089	0.097	0.097	0.090	0.094	0.097	0.109*
	(0.061)	(0.060)	(0.060)	(0.061)	(0.060)	(0.060)	(0.060)
Accountability	0.073	0.070	0.072	0.077	0.073	0.063	0.069
	(0.057)	(0.057)	(0.083)	(0.056)	(0.058)	(0.083)	(0.057)
Claim Experience	-0.018	-0.027	-0.027	-0.020	-0.034	-0.027	-0.033
	(0.069)	(0.068)	(0.068)	(0.069)	(0.070)	(0.068)	(0.068)
Gynaecology	-0.081			-0.067			
	(0.122)			(0.118)			
Defensive Person	0.012	0.016	0.018	0.014	0.018	0.016	0.013
	(0.056)	(0.057)	(0.058)	(0.056)	(0.057)	(0.057)	(0.056)
Critical Patient X Gynaecology	0.057						
	(0.115)						
Critical Patient X Female		-0.008					
		(0.111)					
Critical Patient X Accountability			-0.005				
			(0.115)				
Liability Risk X Gynaecology				0.034			
				(0.122)			
Liability Risk X Female					0.097		
					(0.111)		
Liability Risk X Accountability						0.013	
						(0.108)	
Critical Patient X Liability Risk							-0.141
							(0.110)
Observations	328	328	328	328	328	328	328
Case FE	No	Yes	Yes	No	Yes	Yes	Yes
Order FE	Yes	Yes	Yes	Yes	Yes	Yes	Yes

The table reports OLS results. Heteroskedasticity robust standard errors (in parentheses) are clustered at the participant level. *** *p* < 0.01, ** *p* < 0.05, * *p* < 0.1

### Robustness checks

Table 9 represents multiple robustness checks. In Column (1) and (2), we estimate Eq. (1) by dropping respectively the fastest and slowest 25 per cent of our sample. In that way, we examine whether our results are sensitive to bias from, for example, respondents interrupting the experiment and continuing later (very long respondent time; max = 46 605 s) or respondents not reading and watching videos very thoroughly (very short respondent time; min = 687 s). The regressions on the subsamples give comparable results as for our full sample. By dropping the slowest 25 per cent [Column (2)], however, the coefficient of liability risk does not remain significantly different from zero. This may be declared by the fact that duration time is rightly skewed (skewness = 7.87; median = 1517 s; mean = 2481.70 s). In Column (3), we run a probit regression model instead of OLS. Comparing these results to those of Table 6 demonstrates that our results are robust to model choices.

**Table 9:** Robustness checks

	Dependent Variable: Defensive Treatment		
	Drop 25% Fastest Respondents (1)	Drop 25% Slowest Respondents (2)	Probit Regression (3)
Critical Patient	0.075	0.013	0.207
	(0.059)	(0.073)	(0.190)
Liability Risk	0.163**	0.118	0.441**
	(0.061)	(0.076)	(0.200)
Female	-0.157**	-0.035	-0.509**
	(0.068)	(0.084)	(0.226)
Working Experience > 20y	0.094	0.068	0.327
	(0.072)	(0.061)	(0.205)
Accountability	0.056	0.104	0.248
	(0.060)	(0.069)	(0.191)
Claim Experience	-0.036	0.004	-0.117
	(0.084)	(0.078)	(0.229)
Defensive Person	-0.014	-0.020	0.057
	(0.065)	(0.070)	(0.196)
Observations	245	243	328
Case FE	Yes	Yes	Yes
Order FE	Yes	Yes	Yes

The table reports OLS results (except for Column (3), Column (3) shows probit results). Heteroskedasticity robust standard errors in (parentheses) are clustered at the participant level. *** *p* < 0.01, ** *p* < 0.05, * *p* < 0.1

## Discussion and limitations

This study started with the aim of assessing the impact of patients’ critical attitude on physicians’ intentions to practice defensive medicine. Prior studies examining defensive medicine mainly focused on the effects of tort reforms, insurance premiums, and claim experience, but not on patient characteristics or behaviour. We found that patients’ critical attitude drives physicians to administer approximately 11 percentage points more intensive treatments than expert panels consider medically acceptable. Since this effect dissipates when controlling for physicians’ perceived liability risk, we can conclude that critical patients drive physicians to practice defensive medicine because of a higher perceived liability risk among this group of patients and not, for example, due to critical patients being more persuasive to get specific treatments. This is supported by our design, since we kept the communication of the critical and non-critical patients equal in substance (for example, patients’ symptoms, concerns, and preferences), and only manipulated the way patients verbally express their thoughts and feelings. In sum, physicians who perceive a relatively high liability risk perform more than 17 percentage points more defensive acts. Given the heath care budget of 28–30 billion Euros in Belgium [41], this number of extra procedures (which are not strictly medically necessary) highlights a dramatically inefficient use of public resources.^9^ Furthermore, concerning the gynaecologist cases, we know that worldwide C-section rates are approximately 21 per cent [44], while the World Health Organisation (WHO) recommends C-section rates between 10 and 15 per cent [45]. Our results show that defensive medicine may (in part) declare this overuse. As increasing health care costs are a concern in developed countries [46] and the negative consequences of, for example, C-section overuse are more clear (for example, infections, reduced fertility and chronic childhood diseases) [45], a thorough analysis of which professional relationship between patients and physicians is efficient should be a primary focus of policy makers. To provide policy makers with additional insights, future work should focus on why exactly physicians fear malpractice lawsuits. In 2010, the Belgian government reformed the medical malpractice system from an only-fault to a not-only fault system. Since the reform, cases of alleged medical malpractice can be resolved through a traditional (1) court procedure or (2) settlement agreement, or (3) a procedure managed by the Fund for Medical Accidents (FMA) [47]. One of the main reasons for the reform was the failing compensatory function of the traditional medical malpractice system. The FMA, however, claims to provide better support to patients suffering a medical mishap or error during the resolution process. The FMA can even award compensation on a no-fault basis, though only in cases of severe and abnormal injuries following a medical mishap. At least in theory, this can overcome complex and expensive court procedures. Nevertheless, when reforming the system, policy makers mainly focused on malpractice victim interests and paid little attention to the impact on physicians’ clinical behaviour. For example, liability rules still apply and due to the low-threshold procedures at the FMA, chances to get involved with incident investigations have increased. Since the system is still built on guilt and culpability and procedures at the FMA are long and complex, physicians may be harmed due to 1) less practice time, which results in lower revenues, 2) financial consequences of being sued, 3) reputation damage and 4) moral damage. More research is needed to assess to what extent these specific factors drive physicians to practice defensive medicine.

Moreover, one should investigate other drivers (besides patients’ critical attitude and medical liability rules) of physicians’ perceived liability risk and consequent defensive behaviour. For example, the literature suggests that elderly, poor, and uninsured patients are less likely to file malpractice claims. Future research is required to investigate whether these characteristics also drive physicians’ defensive behaviour. Furthermore, besides performing a more intensive treatment, defensive communication is another form of physicians’ defensive behaviour. That is, physicians may be reluctant to communicate openly about medical incidents out of fear of malpractice claims. An interesting research question for a future study is whether patients’ critical attitude also leads to more defensive communication in practice.

We ensure the high quality of our study results by using an experimental design with strong manipulations and realistic videos, as validated by more than 30 field experts and a thorough literature study. To the best of our knowledge, this has never been done before. Since actual physicians evaluated the videos (instead of medicine students), the results are expected to represent real physician behaviour. However, we measured intentions, not real behaviour. Additionally, our sample only included Flemish gynaecologists and orthopaedists. Further research should address the prevalence of defensive medicine in a real-life context, and among other specialists and in other regions.

## Conclusion

Using a video experiment, we investigated whether patients’ critical attitude drives physicians’ perceived liability risk and consequent defensive behaviour (prescribing more intensive treatment than considered medically needed out of fear of malpractice claims). In particular, 85 gynaecologists/obstetricians and orthopaedists evaluated four videos regarding hypothetical consultations. Half of the physicians was assigned to videos in which the patient poses critical questions. The other half saw exactly the same videos, except that the patient demonstrates a non-critical attitude. We kept all other factors (physician behaviour and communication, patients’ symptoms and preferences, etc.) constant to ensure that patients’ verbal critical attitude drove the treatment effect. Herewith, we overcome omitted variable bias, which is common in existing literature. Our findings indicate that physicians perform significantly more defensive treatment among critical patients because of a higher perceived liability risk. In particular, physicians experiencing a high liability risk perform 17 percentage points more defensive acts (such as unnecessary C-sections, scans, surgeries).

### Supplementary Information

**Additional file 1: Appendix.** 

## Data Availability

The data that support the findings of this study are available on request from the corresponding author L.D. The data are not publicly available due to information that could compromise research participant privacy/consent.

## Footnotes

1 Twenty physicians, four nurses, two health care managers, seven scholars (in the context of patient safety, patient communication and medical liability) and three lawyers.

2 The majority of all specialists in Belgium are male [38].

3 The physicians were informed about the hypothetical nature of the videos in the introduction of the experiment (see Appendix).

4 All treatment options for the various cases can be found in the Appendix.

5 The full questionnaire can be found in the Appendix.

6 Because of randomisation, we ensure the treatment, that is higher liability risk because of a more critical patient, and no other factors, such as payment structure, is driving the treatment effect.

7 Since we got consistent results by using the original Likert scale, we choose to use a dummy for interpretational reasons as described in Table 3.

8 We also ran analyses with original numeric values for physician’s working experience and defensive nature. Since we got consistent results for the coefficients of interest, we choose to use dummy variables for interpretational reasons as described in Table 3. The analyses with original numeric values for physician’s working experience and defensive nature are available upon request from the authors.

9 In the US, the total net costs of defensive medicine are estimated at roughly 2–10 per cent of medical expenditures [42, 43].
