# Supplementary material for: Does patient behaviour drive physicians to practice defensive medicine? Evidence from a video experiment
Source: Health Econ Rev. 2023 Sep 22;13:45. doi: 10.1186/s13561-023-00458-3 (PMC10515032; doi:10.1186/s13561-023-00458-3)
Supplement: Supplementary file 1 — Additional file 1: Appendix. [file 13561_2023_458_MOESM1_ESM.docx]

**Appendix**

Instructions for the doctors

In this study, you assess 4 hypothetical medical situations. For each situation, we first ask you to read a description. Next, you are shown a video about the medical situation and asked to answer some questions regarding this video. We are interested in your personal opinion. In other words, there are no right or wrong answers.

Transcripts of one of the videos

Previous C-section

*Description:* A 30-year-old woman is 38 weeks pregnant with her second child and comes for consultation. Her first child was delivered by caesarean section because of breech presentation. Echo, monitor and clinical examination were completely normal. Baby is now in head position. After a few examinations, the woman comes to the gynaecologist.

*Transcript of the video:*

*Doctor:* Okay, your blood pressure is normal and, on the ultrasound, everything looked okay. The baby is moving a lot you said earlier so that's all positive. The baby now weighs about 3.4 kg and is estimated at 3.6 kg because you have two weeks to go I see, that's a normal weight. He is also doing fine on the monitor. The heart tones have a normal rhythm and we heard them well. As for the rest, your cervix is clearly thinning out already so that's good. The head already seems to be well inside the pelvis, so your baby is already quite well descended. So now the baby is not in breech position, like last time with your first child. You can therefore basically give birth vaginally, unlike last time, when a caesarean section was really necessary.

*Patient:* Okay, that's all good to hear. Suppose I were to try to give birth vaginally now, what are the chances of ending up with a caesarean section eventually?

*Doctor:* Well, that chance is about 10-15%. Like all pregnant women, you have a 1 in 10 chance on top of that. Together, that makes a good 2 in 10. So, in about 75 to 80% of women who have a breech presentation in their first birth, a vaginal birth succeeds in their second birth. We then let nature take its course as much as possible. This means that we allow labour to start spontaneously and check whether dilation is progressing smoothly. However, the course of labour, that is dilation and so on, cannot be compared to that of a woman giving birth to a second child but rather to someone having a child for the first time. We can do a caesarean section at any time during dilation and labour if we notice that dilation is not progressing, if the baby is not descending sufficiently, or the heart tones are not good.

*Non-critical patient:* Oh yes, I can imagine. Eventually, suppose I’ll give birth by C-section, would that be an emergency procedure or how should I prepare for that? For example, in that case, would it be possible for my husband to attend the delivery?

*Critical patient:* If I understand correctly, I still have a 25 per cent chance that I’ll get an emergency C-section and that my husband cannot attend the delivery because I’ll get anaesthesia.

*Doctor:* If we have to proceed to caesarean section because labour is not progressing, we do not immediately speak of an emergency caesarean section. A caesarean section is then indicated but we normally have enough time to proceed to a caesarean section and you do not need to be under anaesthesia so your husband can assist you during labour. He doesn't have to miss a second of that.

*Non-critical patient:* Okay, I can imagine. And would that be dangerous for the baby or wouldn’t you expect any complications?

*Critical patient:* Okay, but can you guarantee that there will be no oxygen deprivation for the baby and that he’ll not suffer? I hope you don't take any risk that could harm me or the baby. I hope you know what you are doing and nothing is going to happen to me or the baby.

*Doctor:* I unfortunately cannot give you 100% guarantee but the chance of oxygen deprivation is very small. If we do find that the baby is in oxygen deprivation, we do call it an emergency caesarean section. You will probably be put under anaesthesia in that case. In many cases, your husband is allowed to attend the birth. We know our job and we’ll do our job. You should not worry about that.

*Non-critical patient:* Okay, and if the labour starts spontaneously, how do you guys see when you should proceed to caesarean section?

*Critical patient:* Yes, but can you see in time when something goes wrong and can you react in time?

*Doctor:* We monitor both you and the baby closely. During labour, you will be permanently under the control of a monitor, which measures the baby's heart rate and also the contraction force of the uterus. If we notice that the baby is struggling or that labour is not progressing sufficiently, we will consider a caesarean section.

*Non-critical patient:* Ah yes, okay. Is there any chance that during labour my scar will open back up?

*Critical patient:* Yes, I do hope you will intervene in time then because suppose my scar ruptures during labour. That chance is pretty high then, isn't it?

*Doctor:* It is true that the caesarean section scar in the uterus weakens and may tear open during labour but that chance is very small. Especially if you did not suffer from your scar during pregnancy. The risk of tearing goes up with certain factors such as a large-weight baby or complications after the first caesarean such as endometritis. But as I mentioned earlier, your baby's weight is perfectly normal and as far as I know, there were no complications with your first caesarean either.

*Non-critical patient:* Okay, if that happened, so it would also be an emergency caesarean section?

*Critical patient:* My neighbour is also a gynaecologist and he told me that such a tear can bleed dramatically so that I could lose my uterus. And then it will surely be an emergency caesarean section. I can assume that's not going to happen to me, right?

*Doctor:* Yes, that's right. If we see that your scar is starting to tear, we need to act quickly.

*Non-critical patient:* Okay, I understand. You probably have experience with this.

*Critical patient:* But do you know how to respond? Do you already have experience with such situations?

*Doctor:* Due to the rare occurrence of uterine rupture, no one really has much experience with it. But, you will be followed 24h/24 by a team of midwives, gynaecologists and anaesthetists and at any time we can intervene.

*Non-critical patient:* Okay, thanks for the clarification. I am somewhat reassured anyway by what you told me today.

*Critical patient:* Yes, but I really do not want anything serious to happen to me or my baby.

*Doctor:* You have to think that the chances of something serious happening are many times smaller than the chances of everything going well.

Treatment options for the scenarios

Previous C-section

- Schedule a caesarean section
- Waiting for spontaneous labour until 40 weeks and then possibly considering whether induction or caesarean section is necessary
- Inducing labour artificially
- Other:

Unclear ultrasound

- Waiting for the next ultrasound
- Scheduling an early ultrasound
- Referring the patient to a specialist for an extended ultrasound
- Other:

IVF baby

- Schedule a caesarean section
- Waiting for spontaneous labour until 40 weeks and then possibly considering whether induction or caesarean section is necessary
- Inducing labour artificially
- Other:

Gestational diabetes

- Schedule a caesarean section
- Waiting for spontaneous labour until 40 weeks and then possibly considering whether induction or caesarean section is necessary
- Inducing labour artificially
- Other:

Elbow fracture

- Continue conservative treatment
- Operate now
- Referral to tertiary centre
- Other:

Ligament tear

- Waiting 3 months + wearing brace + physiotherapy
- Schedule an operation
- Referral to tertiary centre
- Other:

Scaphoid fracture

- Schedule NMR scan
- Schedule CT scan
- Doing nothing
- Placing a cast
- Schedule surgery
- Other:

Shoulder fracture

- Continue conservative treatment
- Scheduling an operation
- Referral to tertiary centre
- Other:

Questionnaire

*After each video*

Which of the following actions would you take if you were the doctor in this specific situation?

(Choice list depending on video, see Section 9.3)

What is the probability that the patient in the scenario would take further action against the doctor if problems or complications arise as a result of the medical treatment?

- Very unlikely
- Unlikely
- Rather unlikely
- Neither unlikely nor likely
- Rather likely
- Likely
- Very likely

To what extent do you agree with the following statements about the patient in the video?

- The patient has confidence in the doctor's skills
- The patient has prior knowledge of the possible risks and complications
- The patient anticipates the facts
- The patient is suspicious
- The patient is critical
- The patient is anxious
- The patient is concerned
- The patient is friendly
- The patient is polite
- The patient is calm
  - Completely disagree
  - Disagree
  - Rather disagree
  - Neither disagree/agree
  - Rather agree
  - Agree
  - Completely agree

To what extent do you agree with the following statements about the doctor in the video?

- The doctor is experienced
- The doctor is competent
- The doctor inspires confidence
- The doctor takes time for the patient
- The doctor handles the consultation well
- The doctor gives the right information to the patient
- The doctor gives sufficient information to the patient
  - Completely disagree
  - Disagree
  - Rather disagree
  - Neither disagree/agree
  - Rather agree
  - Agree
  - Completely agree

*End of questionnaire*

What gender do you identify with?

- Male
- Female
- X
- I wish to not answer this question

How long have you been working as a doctor?

- Less than 1 year
- 1 to 5 years
- 6 to 10 years
- 11 to 20 years
- 21 to 30 years
- More than 30 years

As a doctor, are you accountable to a superior/colleague for the actions you take?

- Yes
- No

In your career as a doctor, were any medical liability claims (e.g. civil/criminal lawsuit, proceedings at the FMO) ever brought against you personally?

- Yes
- No

To what extent do you agree with the following statements? To reduce my risk of medical incidents

- I reduce the number of patient contacts
- I treat fewer high-risk patients
- I perform fewer high-risk medical treatments
- I work fewer hours
- I retire earlier
- I change to a lower-risk specialty
- I refer more patients to confirm my diagnosis (second opinion)
- I prescribe more medication
- I suggest more diagnostic tests to confirm a diagnosis
- I suggest more invasive tests to confirm a diagnosis
  - Completely not
  - Not
  - Rather not
  - Neither not/yes
  - Rather yes
  - Yes
  - Completely yes
